# Uncovering Shortcomings and Deficiencies in the Systematic Review and Meta-Analysis on Ultraprocessed Food Consumption and Human Health

**DOI:** 10.1016/j.advnut.2024.100201

**Published:** 2024-03-28

**Authors:** Janett Barbaresko, Alexander Lang, Sabrina Schlesinger

**Affiliations:** a Institute for Biometrics and Epidemiology, German Diabetes Center, Leibniz Center for Diabetes Research at Heinrich Heine University, Düsseldorf, Germany; b German Center for Diabetes Research (DZD), Partner Düsseldorf, Munich-Neuherberg, Germany

Dear Editor:

We read with great interest the systematic review and meta-analysis by Vitale et al. [1] investigating the consumption of ultraprocessed foods and several health outcomes. The topic of ultraprocessed food consumption has become highly prominent during the last years, making it particularly timely for discussion. However, we found several methodological issues and flaws in this systematic review and meta-analysis that need to be revised. First of all, the authors refer to the incidence of diabetes, hypertension, hypertriglyceridemia, low high-density lipoprotein-cholesterol, and obesity in their meta-analysis. However, they included different study designs and summarized results from cross-sectional and prospective cohort studies, which could affect the interpretation of the results. Furthermore, it is notable that the authors did not present confounders considered nor did they adequately assess risk of bias in the primary studies, and there is no discussion on potential confounding. Vitale et al. [1] used the Newcastle–Ottawa Scale (NOS), which underestimates risk of bias and thus, overestimates the study quality [2]. Additionally, the authors did not transparently present the criteria for the judgment of single items of the NOS, and the consideration of confounding remains unclear. Consequently, the interpretation of the findings should be approached with caution.

Moreover, although the title of the publication by Vitale et al. [1] implies that many outcomes will be presented, we were surprised that the authors solely selected type 2 diabetes and some cardiovascular disease risk markers, such as hypertension, hypertriglyceridemia, and obesity. These outcomes have already been covered in previous systematic reviews and meta-analyses [[3], [4], [5], [6]]. Another major concern is the systematic literature search, including the incompleteness of the meta-analyses and the noninclusion of relevant articles. For example, 3 large cohort studies (Nurses’ Health Study, Nurses’ Health Study II, and Health Professional Follow-up Study) are missing in the meta-analysis on the consumption of ultraprocessed food and risk of type 2 diabetes [3]. Regarding hypertension, a previous meta-analysis on this topic was published last year [4]. The underlying report by Vitale et al. [1] identified 1 new primary study, but it is unclear why there is a discrepancy of 5 studies missing in their meta-analysis compared with the previous systematic review [4]. For low high-density lipoprotein and hypertriglyceridemia, Vitale et al. [1] found 3 new articles but missed 2 studies previously included in another systematic review and meta-analysis [5]. As part of the literature search process, it is common practice to screen reference lists of relevant systematic reviews to identify additional relevant articles, ensuring a comprehensive and complete overview of the currently available evidence. In addition, the authors did not include a list of excluded studies with reasons (as postulated in the PRISMA 2020 statement) and thus, the systematic search process is not transparent. Furthermore, with regard to the literature-screening process, the authors stated in the flow chart that they excluded 174 studies because they did not retrieve the full texts. This introduces a high risk of bias, and the authors should put effort into accessing these articles.

With regard to the data extraction, we found several inconsistencies between the extracted data and the data shown in the original studies. Here are some examples: first, the meta-analysis on obesity does not exclusively include studies on obesity (BMI ≥ 30 kg/m^2^) but also studies on overweight (BMI ≥ 25 kg/m^2^), which could affect the interpretation of the findings. Second, in the meta-analysis on high consumption compared with low consumption of ultraprocessed food and health outcomes, Vitale et al. [1] included risk estimates that referred to a dose–response estimate (risk ratio per 10% increment in ultraprocessed food intake). Third, in the meta-analysis on obesity, the authors only included the data on females and not on men for 1 primary study, introducing selection bias. Fourth, the number of participants and cases were not correct for several studies.

Finally, the authors decided to use the NutriGrade tool to assess the quality of evidence. However, it appears that there was a misalignment in its application. Instead of evaluating the certainty of meta-evidence for the overall associations, the authors rated each included study separately and calculated a mean for each outcome, which is not the correct approach.

In conclusion, this report [1] requires extensive revision. This should include an unbiased literature search, accurate and unselective data extraction, an adequate assessment of risk of bias with a particular focus on potential confounders, and a correct evaluation of the certainty of evidence. In addition, methodological concepts, such as the distinction between prevalence and incidence, are crucial for understanding the data on ultraprocessed food consumption and risk of health outcomes.

## Conflict of interest

The authors report no conflicts of interest.
